# Arbuscular mycorrhizal fungal families and exploration‐based guilds exhibit distinct responses to long‐term N, P and K deficiencies and imbalances

**DOI:** 10.1111/nph.70969

**Published:** 2026-03-02

**Authors:** Kian Jenab, Lauren Alteio, Ksenia Guseva, Stefan Gorka, Sean Darcy, Lucia Fuchslueger, Alberto Canarini, Victoria Martin, Julia Wiesenbauer, Felix Spiegel, Bruna Imai, Hannes Schmidt, Karin Hage‐Ahmed, Erich M. Pötsch, Andreas Richter, Jan Jansa, Christina Kaiser

**Affiliations:** ^1^ Division of Terrestrial Ecosystem Research, Centre for Microbiology and Environmental Systems Science University of Vienna Vienna 1030 Austria; ^2^ Doctoral School in Microbiology and Environmental Science University of Vienna Vienna 1030 Austria; ^3^ Austrian Competence Centre for Feed and Food Quality, Safety and Innovation, FFoQSI GmbH Technopark 1‐D Tulln 3430 Austria; ^4^ Environment and Climate Hub University of Vienna Vienna 1090 Austria; ^5^ Department of Biological, Geological and Environmental Sciences University of Bologna Bologna 40126 Italy; ^6^ Centre for Food Science and Veterinary Public Health, Clinical Department for Farm Animals and Food System Science University of Veterinary Medicine Vienna 1210 Austria; ^7^ Department of Agricultural Sciences, Institute of Plant Protection BOKU University Vienna 1190 Austria; ^8^ Agricultural Research and Education Centre, Raumberg‐Gumpenstein Irdning 8952 Austria; ^9^ Laboratory of Fungal Biology, Institute of Microbiology of the Czech Academy of Sciences Prague 14200 Czech Republic

**Keywords:** AMF abundance and community composition, ancestral, arbuscular mycorrhizal fungi, edaphophilic, exploration‐based functional guild, long‐term nutrient deficiencies and imbalances, managed grassland, rhizophilic

## Abstract

- Many agroecosystems face nitrogen (N), phosphorus (P) or potassium (K) deficiencies due to imbalanced or insufficient nutrient replenishment after biomass harvest. How this affects the symbiosis between plants and arbuscular mycorrhizal fungi (AMF) and the abundance of exploration‐based AMF guilds (rhizophilic, edaphophilic and ancestral) remains largely unknown.
- We studied a 70‐yr nutrient deficiency experiment in a managed grassland in central Austria, where aboveground biomass was harvested three times annually. N, P and K were fully, partially or not replenished, causing long‐term nutrient deficiencies and imbalances. We analysed AMF communities in soil and roots by DNA/RNA amplicon sequencing and fatty acid biomarkers, alongside soil and plant community properties.
- Soil AMF communities were affected by N and P deficiencies, while root AMF communities were most susceptible to K deficiency, showing up to 50% biomass reduction, particularly when N was abundant. We observed a shift from rhizophilic to ancestral guilds under P deficiency in soil, and under K deficiency in roots. Families within each guild, particularly ancestral, showed differential responses, indicating complementary nutrient specializations at the family level.
- Our findings underscore the previously unrecognized role of K deficiency in AMF symbiosis and suggest the existence of nutrient‐related functional subgroups within exploration‐based AMF guilds.

## Introduction

Arbuscular mycorrhizal fungi (AMF) are among the oldest and most important plant root symbionts (Humphreys *et al*., 2010; Brundrett & Tedersoo, 2018), forming associations with most plant species in croplands and managed grasslands. They provide essential soil nutrients such as nitrogen (N), phosphorus (P) and potassium (K) to host plants (Smith & Smith, 2011; Berruti *et al*., 2016; Igiehon & Babalola, 2017), along with other benefits such as pathogen protection (Borowicz, 2001) and improved soil structure (Rillig & Mummey, 2006).

AMF are known to be sensitive to increased soil nutrient availability from fertilization (Johnson *et al*., 2015), yet their responses to nutrient deficiencies and imbalances are less clear. Although many agricultural soils in industrial regions are overfertilized, a substantial proportion of global agricultural soils experiences severe deficits because fertilization fails to replace nutrients removed at crop harvest (Manning, 2012; Ludemann *et al*., 2024). As of 2000, net annual losses of *c*. 18.7 N, 5.1 P and 38.8 K (kg ha^−1^ yr^−1^) affected 59, 85 and 90% of harvested area world‐wide, respectively (Tan *et al*., 2005). Imbalances are particularly pronounced for K: Insufficient K replenishment relative to N and P occurs in *c*. 20% of global agroecosystems, particularly in Africa and China (Tan *et al*., 2012; Zörb *et al*., 2014), a trend exacerbated by rising K fertilizer costs (Brownlie *et al*., 2024). In parallel, anthropogenic increases in atmospheric N deposition elevate N : P and N : K ratios across ecosystems, altering soil nutrient stoichiometry (Peñuelas *et al*., 2013; Lu & Tian, 2017).

AMF are particularly relevant in low‐fertilization agroecosystems. Under low nutrient availabilities, AMF are pivotal because their extraradical hyphal networks can access inorganic nutrients (in particular P) more efficiently than roots (Smith & Smith, 2011). Conversely, when nutrients are plentiful, plants typically reduce support for AMF (Hoeksema *et al*., 2010). For example, high P fertilization commonly reduces root colonization (Propster & Johnson, 2015; Liu *et al*., 2016; Williams *et al*., 2017). Beyond absolute amounts, however, also relative proportions of available nutrients matter (Johnson, 2010; Johnson *et al*., 2015): AMF biomass tends to increase with N addition when P is limiting but declines once soil P exceeds a threshold (Johnson *et al*., 2015; Jiang *et al*., 2018; Han *et al*., 2020). In contrast to inorganic fertilizers, organic fertilizers often enhance AMF growth, possibly due to slower nutrient release (Cavagnaro, 2015; Yang *et al*., 2018). Although extensive research has been carried out investigating AMF responses to P or N availability, AMF responses to absolute or relative changes in K availability remain largely unexplored.

Nutrient shortages and imbalances can also influence AMF indirectly through ecosystem feedbacks that unfold over time. Nutrient‐driven shifts in vegetation or edaphic properties (e.g. pH, water or organic matter content (Jamiołkowska *et al*., 2018)) further affect AMF communities. Particularly, soil pH is a key determinant of AMF distribution and abundance (Clark, 1997; Van Aarle *et al*., 2002; Van Geel *et al*., 2018; Davison *et al*., 2021). In turn, changing AMF communities have the potential to alter ecosystem properties by affecting plant communities through effects on plant competitiveness, affecting soil physical structure and building soil C stocks via their mycelia (Rillig & Mummey, 2006; Wang *et al*., 2016; Hawkins *et al*., 2023).

Such ecosystem feedbacks of nutrient‐induced changes in AMF communities could be mediated by AMF functional traits. The proportion and extent of AMF mycelia extending into soil, for example, is a trait of potential ecosystem relevance, which is phylogenetically conserved at the family level (Hart & Reader, 2002; Powell *et al*., 2009). Accordingly, AMF families have been grouped into three exploration‐based guilds: edaphophilic, with extensive extraradical (i.e. outside plant roots in the soil) but limited intraradical (i.e. inside plant roots) mycelia (e.g. Gigasporaceae); rhizophilic, with abundant intraradical but sparse extraradical mycelium (e.g. Glomeraceae); and ancestral, with low biomass both intra‐ and extraradically (Maherali & Klironomos, 2007; Phillips *et al*., 2019; Weber *et al*., 2019). Mycelial architecture and specific taxon‐ and family‐level observations suggest that these guilds differ functionally (Maherali & Klironomos, 2007; Powell *et al*., 2009; Treseder *et al*., 2018; Weber *et al*., 2019): Edaphophilic taxa are thought to better support plant nutrient uptake and strengthen soil C sinks due to their larger soil‐based mycelium, whereas rhizophilic taxa may be more effective in pathogen defense and conferring stress tolerance (Chagnon *et al*., 2013; Weber *et al*., 2019), although members of Gigasporaceae can also excel under biotic stress in some cases (Marro *et al*., 2022). Thus, nutrient‐driven shifts among AMF guilds could meaningfully affect ecosystem functions, such as plant nutrition and soil C sequestration.

Despite the potential relevance of AMF in agroecosystems, long‐term AMF responses to increasing nutrient deficiencies and imbalances remain insufficiently understood. Only a few studies have investigated long‐term effects of nutrient deficiencies on AMF abundance and community composition in managed grasslands or croplands (Antunes *et al*., 2012; Qin *et al*., 2015; Heyburn *et al*., 2017; Zheng *et al*., 2018). Responses of exploration‐based guilds are largely unexplored, and most work has centred on P or N, leaving K deficiency and imbalances as important knowledge gaps.

Here, we investigate how > 70 yr of N, P and K deficiencies and imbalances shape AMF communities in a managed grassland. We ask whether long‐term single and combined deficiencies of N, P and K influence AMF composition and abundance in soil and roots, and whether these changes manifest in changes in exploration‐trait‐based functional guilds. We further test whether AMF responses are linked to changes in plant communities and soil properties. To address these questions, we used a nutrient deficiency experiment launched in 1946 in a permanent grassland site in Austria. Treatments included factorial deficiencies of N, P and K and regular liming to manipulate pH. We quantified intra‐ and extraradical AMF mycelia from soil and root samples, and profiled AMF communities molecularly to assign exploration‐based guilds. Plant community composition and soil abiotic factors are assessed to identify potential links between these parameters and changes in AMF communities.

## Materials and Methods

### Study site

The study was conducted at a long‐term nutrient deficiency experimental grassland site of the Agricultural Research and Education Centre Raumberg‐Gumpenstein in Admont, Styria, Austria (47°34′58″N, 14°27′02″E; 635 m asl). This grassland field experiment was established in 1946 on a hay meadow, with a three‐cut per year management regime since start. According to the WRB soil classification system, the soil is a Gleyic Fluvic Dystric Cambisol with no supplementary qualifiers (IUSS Working Group WRB, 2015). The long‐term mean annual precipitation is 1227 mm, and the mean annual temperature is 6.8°C.

### Nutrient deficiency experiment

The experiment includes 24 nutrient‐fertilization treatments (4 replicates each; 2.9 m × 7.1 m) in a randomized block design, of which 14 treatments were selected for our study (Supporting Information Fig. S1). The treatments comprise variations of inorganic (P, N and K) or organic (solid manure and liquid slurry) fertilization. Furthermore, some plots receive lime alongside inorganic fertilizers to examine the effect of pH independent of nutrient deficiencies. Note that for the rest of the manuscript, we refer to ‘inorganic treatments’ as those only receiving inorganic or no fertilizers at all (excluding the lime treatments), whereas ‘all treatments’ encompass inorganically and organically fertilized as well as limed plots. N is applied as calcium ammonium nitrate (NH_4_NO_3_ + CaCO_3_), at 80 kg N ha^−1^ yr^−1^, split between spring and after the first cut. Calcium ammonium nitrate is a widely used inorganic fertilizer, which contains CaCO_3_ to buffer the acidifying effect of NH_4_NO_3_, thereby having a near‐neutral effect on soil pH. P was applied as Thomasphosphate ((CaO)_5_ P_2_O_5_ SiO_2_) from 1946 to 1997, and as hyperphosphate (Ca(H_2_PO_4_)_2_) thereafter at 35 kg P ha^−1^ yr^−1^, while K fertilization as KCl at 100 kg K ha^−1^ yr^−1^, both in autumn. Some variants received additionally mixed lime (CaCO_3_ + MgCO_3_) at 928 kg Ca ha^−1^ every third year in autumn (referred to as ‘lime treatments’). Solid manure (37% N, 12% P, 51% K) and liquid slurry (35% N, 1% P, 64% K) are added at 15 t ha^−1^ (in autumn) and 40 t ha^−1^ (split between spring and postcut), respectively. Organic treatments provide more balanced nutrient inputs and reflect conventional fertilization practices in agroecosystems. While all three nutrients are provided in the fully fertilized treatment and the organic treatments, one or more nutrients are missing in the other treatments. Consequently, while aboveground plant biomass and nutrients have been removed consistently, only part of the nutrients have been replenished by fertilization. Additionally, the unfertilized control treatment has never received any fertilizer while being mowed three times a year since 1946. This practice has led to different types of long‐term nutrient deficiencies in this grassland.

### Sample collection

In July 2019, soil samples were collected from 55 plots, comprising 14 treatments with four replicate plots each, except for the ‘lime only’ treatment, which was sampled from three replicate plots. The samples were taken to 9.5 cm depth using 2‐cm‐diameter corers. For each plot, 8 to 11 randomly distributed cores were combined into an *c*. 300 g composite sample. Samples were sieved to 2 mm, and roots were separated from the soil. Fine roots were washed and cleaned. Subsamples for DNA and RNA extraction from root and soil samples were frozen in liquid N_2_ and stored at −80°C. After lyophilization, root samples were ground in a cryomill with liquid N_2_, and both soil and root samples were stored at −20°C for further analysis.

### Soil characterization

Soil pH (H_2_O) was measured in 1 : 5 (w/v) soil slurry. Soil water content was measured gravimetrically by weighing soil before and after drying at 105°C for 24 h. For soil total C and N content, soil aliquots were dried at 60°C for 72 h, ground and measured by an elemental analyser coupled to isotope‐ratio mass‐spectrometry (EA‐IRMS) (EA 1110; CE Instruments, Milan, Italy), coupled to a Finnigan MAT Delta Plus IRMS (ThermoFisher Scientific, Waltham, MA, USA).

Plant‐available inorganic phosphate was measured photometrically by the phosphomolybdate blue reaction (Schinner *et al*., 1996), in 0.5 M NaHCO_3_ extracts, with or without prior alkaline persulfate oxidation to estimate total dissolved P and dissolved inorganic P (Doyle *et al*., 2004). Ammonium was quantified in 1 M KCl soil extracts spectrophotometrically by Berthelot reaction (Tecan Infinite M200 Fluorimeter, Grödig, Austria) (Kandeler & Gerber, 1988; Hood‐Nowotny *et al*., 2010). Nitrate was measured in 1 M KCl extracts by the VCl_3_ reduction and Griess reaction (Miranda *et al*., 2001; Hood‐Nowotny *et al*., 2010). Soil total free amino acids were determined fluorometrically based on Jones *et al*. (2002). Total dissolved N and dissolved organic C were measured in 1 M KCl soil extracts by TOC/TN‐analyzer (TOC‐V CPH/TMN‐1, Shimadzu, Japan), and dissolved organic N was calculated by subtracting ammonium and nitrate from total dissolved N.

Microbial C, N and P were measured using chloroform fumigation‐extraction (Vance *et al*., 1987), with modifications. Four grams of soil from each plot replicate was weighed in two containers. To the first set of containers, 30 ml of 1 M KCl solution (for C and N) or 0.5 M NaHCO_3_ (for P) was added and shaken for 1 h before filtering through ash‐free cellulose filters. The extracts were kept frozen at −20°C for later analyses. The second set was fumigated with chloroform for 48 h in darkness, then extracted similarly. Dissolved organic C, total dissolved N and P were measured in the extracts as described previously. Microbial biomass C was calculated by dividing fumigated and nonfumigated sample differences by 0.45, and microbial biomass N by 0.54; no factor was used for microbial biomass P (McDonald *et al*., 1998).

### Plant community composition

The most recent measurements of plant community composition (2015) and biomass (2018) were used, as they are the closest available measurements to our sampling date, ensuring the highest possible relevance to our study. Although we cannot rule out that plant community composition may have slightly shifted within a few years, we still think that this dataset holds valuable information, as plant community composition tends to remain relatively stable over decades; for example, a Central European dry grassland showed no directional change in species composition over 90 yr, despite interannual fluctuations (Fischer *et al*., 2020).

The plant community composition was determined in 2015 using the modified method of Braun‐Blanquet (1951) described in Peratoner & Pötsch (2019). The plant species were identified taxonomically following Fischer *et al*. (2008). Plots were harvested in 2018 using a motorized bar mower, and green mass yield was determined in the field. Representative samples were taken from each plot for dry matter content and forage quality analysis using standardized methods by VDLUFA (1976) and ALVA (1983).

### Neutral fatty acid biomarkers as proxy for AMF biomass

Neutral fatty acids (NLFAs) in soil and root samples were evaluated by the procedure described in Gorka *et al*. (2023). Total lipids were extracted with chloroform, methanol and 0.15 M citrate buffer (pH = 4; v/v/v = 1 : 2 : 0.8) (Bligh & Dyer, 1959). Neutral lipids were isolated by elution from 96‐well silica plates (Strata SI‐1 Silica, 55 μm, 70 Å, 50 mg per well; Phenomenex, Torrance, CA, USA) with chloroform containing 2% ethanol (Buyer & Sasser, 2012; Drijber & Jeske, 2019; Gorka *et al*., 2023). Subsequently, NLFAs were released from the lipids and transmethylated by alkaline methanolysis. Methyl‐nonadecanoate (10 μg sample^−1^) was added as an internal standard for quantification of fatty acids by GC (7890B Agilent Technologies, Santa Clara, CA, USA) coupled to a TOF‐MS (Pegasus BT, LECO, USA). NLFA 16:1ω5 was identified by comparison of chromatographic retention times to external fatty acid methyl ester standards (37 Component FAME mix, and BAME CP mix; Merck‐Supelco, Darmstadt, Germany), and by comparison with the National Institute of Standards and Technology (NIST) library of mass spectra. We used NLFA 16:1ω5 as a proxy for soil and root AMF biomass (Kaiser *et al*., 2015).

### AMF amplicon sequencing

AMF community composition was assessed by 18S rRNA gene amplicon sequencing using both RNA (cDNA) and DNA following Bukovská *et al*. (2021), using WANDA (CAGCCGCGGTAATTCCAGCT) and AML2 (GAACCCAAACACTTTGGTTTCC) primers. For RNA‐based amplicon sequencing, total nucleic acids were treated with DNAse and reverse‐transcribed to cDNA using SuperScript™ III (Invitrogen).

We conducted RNA and DNA amplicon sequencing in soil samples, while only DNA amplicon sequencing in root samples. In soil samples, total nucleic acids were extracted by phenol–chloroform procedure (Angel *et al*., 2012), whereas Qiagen DNeasy Plant Mini Kit was used to extract DNA from root samples. The amplicons were dually indexed with Nextera XT indexes and sequenced on Illumina MiSeq 2 × 300 platform (Nilsson *et al*., 2019; Bukovská *et al*., 2021). We used the SEED software and external software packages to process sequencing results (Větrovský & Baldrian, 2013; Jansa *et al*., 2020). A minimum of 20‐bp overlap was considered to pair raw sequence reads, and sequences with average quality scores below 30 were eliminated (Bukovská *et al*., 2021). Primers and chimeras were removed from sequences, and reads were clustered at a 97% similarity threshold into operational taxonomic units (OTUs). These OTUs were identified using the SILVA database, retaining only Glomeromycota OTUs (Bukovská *et al*., 2021). Relative abundances of OTUs per sample were merged at genus level. AMF families were categorized based on their known growth traits (hyphae allocation in soil and roots) into three main guilds: rhizophilic, edaphophilic and ancestral (Parniske, 2008; Powell *et al*., 2009; Weber *et al*., 2019).

DNA and RNA amplicon sequencing showed a comparable pattern of soil AMF communities (Fig. S2). However, DNA amplicon sequencing yielded insufficient sequencing depths for AMF communities in soil samples, as WANDA primer also amplified tardigrades, nematodes and other invertebrates in soil. Due to higher sequencing depth yield, we used RNA amplicon sequencing of the 18S rRNA gene to assess soil AMF communities.

### Statistical analysis

Statistical analyses were conducted in R (v4.3.3), and plots generated using the package ggplot2 (v.3.4.4) (Wickham, 2016). We tested whether N, P and K additions affected AMF and plant biomass as well as environmental variables in a full factorial way using a three‐way analysis of variance (ANOVA) on the source data after checking normality and variance homogeneity. For non‐normally distributed data, we applied transformations (square root, logarithmic or exponential) to achieve normality. To assess lime application effects on AMF biomass, we performed a two‐way ANOVA for lime‐treated plots with lime application and inorganic nutrient fertilization as factors. For plots receiving organic fertilizers, we conducted a two‐way ANOVA with solid manure and liquid slurry applications as factors.

Multivariate analyses used the vegan package (v.2.6‐2) (Oksanen *et al*., 2024). We analysed long‐term nutrient deficiency influences on soil and root AMF and plant communities by correspondence analysis (CA), suitable for unimodal species distributions in ecological datasets (Ter Braak, 1985), and across treatments. CA1 scores represented AMF and plant community composition, as this axis explained most variation. We applied permutational multivariate analysis of variance (PERMANOVA) to assess treatment effects on community compositions. We assessed soil and root AMF relationships with plant community compositions using the Mantel and partial Mantel test, based on Bray–Curtis dissimilarities for AMF and plant community data, and Euclidean distances for edaphic factors, and Spearman's rank correlation coefficient. Associations between AMF abundance and community composition, and environmental variables were evaluated using Spearman's correlation for normally and non‐normally distributed variables. Canonical CA (CCA) was used to examine the influence of environmental factors on AMF and plant community compositions.

## Results

### Long‐term fertilization regimes altered soil nutrient contents and pH

Over 70 yr of annual biomass harvest without replenishment of N, P or K has significantly altered availabilities of these elements in soil. Compared with fully fertilized control plots, long‐term absence of N fertilization significantly reduced total dissolved N as well as dissolved inorganic N pools (ammonium and nitrate), but it increased at the same time dissolved organic N (*P* < 0.001; Fig. S3d; Table S1). It also reduced plant biomass, increased soil pH and inorganic P (Fig. S3a,f; Table S1).

**Fig. 1:**
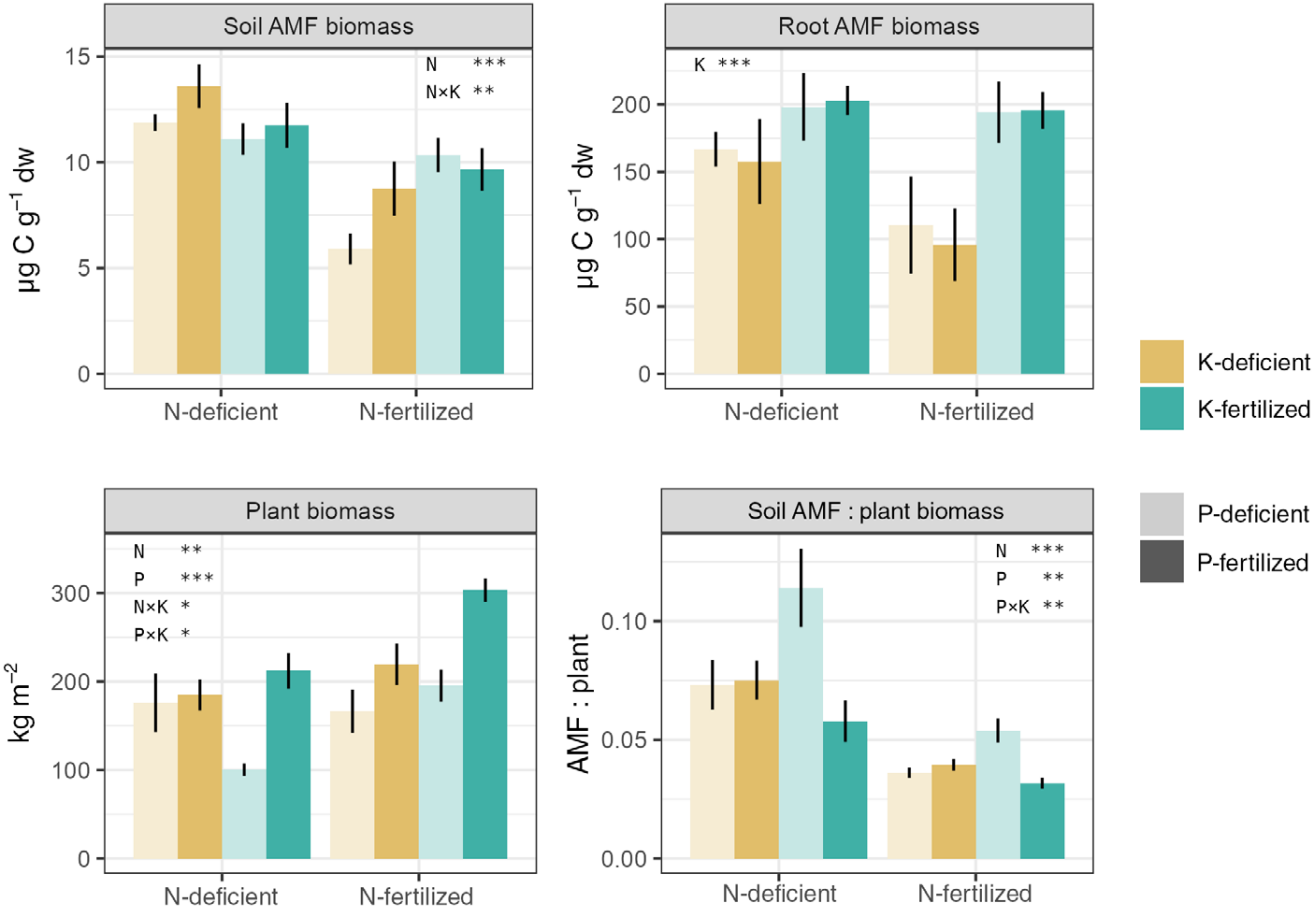
Long‐term deficiencies of nitrogen (N), phosphorus (P) or potassium (K) affect arbuscular mycorrhizal fungi (AMF) biomass in soil and roots, and plant biomass. Soil AMF biomass increased with N deficiency, while root AMF biomass decreased with K deficiency (*P* < 0.05, analysis of variance (ANOVA); Table 1). The panels show soil (i.e. extraradical) and root (i.e. intraradical) AMF biomass quantified using the neutral lipid fatty acid 16:1ω5 as a biomarker, as well as aboveground plant biomass and soil AMF biomass per plant biomass unit. *X*‐axes depict N treatments (left bars, N‐deficient; right bars, N‐fertilized). Colours indicate K treatments (ochre, K‐deficient; cyan, K‐fertilized). Colour transparency indicates P treatments (light colours, P‐deficient; dark colours, P‐fertilized). Bars represent mean ± SE of the mean (*n* = 4). Statistical significance from the three‐way ANOVA is reported within each panel: *, *P* < 0.05; **, *P* < 0.01; ***, *P* < 0.001 (Table 1).

Long‐term absence of P inputs significantly reduced all measured P pools (dissolved organic P, inorganic P and microbial P), plant biomass and soil pH (Figs 1, S3a,e,f; Table S1), but increased C pools (dissolved organic C and soil C content) and most N pools such as soil N content, total dissolved N, total free amino acids and ammonium (Fig. S3a; Table S1).

Lack of potassium fertilization significantly increased soil pH and water content (Fig. S3a,b; Table S1). While we did not measure soil K, we refer to Pavlů *et al*. (2022), who report soil plant‐available K, P, Ca and Mg from the same long‐term nutrient‐deficient experiment. Their findings show that available K nearly doubled in K‐only fertilized treatments (238.8 mg kg^−1^) compared with unfertilized controls (*c*. 98 mg kg^−1^). However, K fertilization significantly enhanced available K only when N and P were deficient, as higher plant biomass production in N and P plots likely offset soil K accumulation (Pavlů *et al*., 2022).

### AMF biomass was linked to N and P in soils, but to K in roots

N, P and K deficiencies, caused by the absence of respective fertilizer inputs, had strong and distinct effects on AMF biomass (using 16:1ω5 NLFA as a proxy) (Fig. 1; Table 1). The responses of AMF biomass in roots and soil (intra‐ and extraradical mycelia, respectively) were driven by different element deficiencies. While N deficiency significantly increased AMF biomass in soil (*F* = 27.51, *P* < 0.001), its effect on AMF biomass in roots was less pronounced (*F* = 4.13, *P* = 0.054; Fig. 1; Table 1). P deficiency had a weak negative influence on AMF biomass in soil (*F* = 3.04, *P* = 0.09), but no effect on AMF in roots (Fig. 1; Table 1). However, P deficiency significantly increased soil AMF biomass per unit of plant biomass, particularly under K fertilization (*F* = 27.51, *P* = 0.005; Fig. 1; Table 1). We also observed a significant interaction between N and K effects on soil AMF biomass (*F* = 9.37, *P* < 0.01; Table 1), with the lowest values in N‐fertilized plots lacking both P and K (Fig. 1). By contrast, K deficiency had the most impact on root AMF biomass and significantly diminished root AMF biomass (*F* = 13.35, *P* < 0.01; Fig. 1; Table 1).

**Table 1:** Effects of long‐term nitrogen (N), phosphorus (P) or potassium (K) deficiencies on soil and root arbuscular mycorrhizal fungi (AMF) biomass, families and guilds in inorganic treatments.

	Relative abundance	−N	−P	−K	−N*−P	−N*−K	−P*−K	−N*−P*−K
Soil AMF								
AMF biomass		0.000***↑	0.094^.^↓	0.310	0.935	0.005**	0.088^.^	0.357
AMF : plant ratio		0.000***↑	0.005**↑	0.170	0.149	**0.575**	0.002**	0.182
Community composition		0.000***	0.000***	0.012*				
Rhizophilic guild	0.52 ± 0.03	0.653	0.000***↓	0.040*↓	0.027*	0.990	0.161	0.201
Claroideoglomeraceae	0.05 ± 0.01	0.442	0.150	0.033*↑	0.591	0.071^.^	0.333	0.324
Glomeraceae	0.34 ± 0.03	0.146	0.830	0.009**↓	0.825	0.499	0.053^.^	0.554
Paraglomeraceae	0.13 ± 0.03	0.255	0.000***↓	0.523	0.037*	0.795	0.441	0.395
Edaphophilic guild	0.07 ± 0.01	0.358	0.327	0.429	0.431	0.817	0.252	0.296
Diversisporaceae	0.02 ± 0.01	0.091^.^↑	0.238	0.007**↑	0.673	0.114	0.476	0.488
Gigasporaceae	0.05 ± 0.01	0.046*↓	0.071^.^↑	0.579	0.231	0.571	0.288	0.345
Ancestral guild	0.41 ± 0.03	0.964	0.000***↑	0.106	0.076^.^	0.895	0.057^.^	0.082^.^
Acaulosporaceae	0.23 ± 0.02	0.001**↑	0.388	0.382	0.434	0.061^.^	0.353	0.991
Ambisporaceae	0.14 ± 0.02	0.000***↓	0.000***↑	0.010*↑	0.002**	0.015*	0.210	0.016*
Archaeosporaceae	0.04 ± 0.01	0.080^.^↓	0.148	0.000***↑	0.035*	0.169	0.032*	0.178
Root AMF								
AMF biomass		0.054^.^↑	0.661	0.001**↓	0.841	0.146	0.672	0.978
Community composition		0.105	0.225	0.004**				
Rhizophilic guild	0.91 ± 0.02	0.143	0.505	0.000***↓	0.100	0.610	0.523	0.563
Claroideoglomeraceae	0.03 ± 0.00	0.058^.^↓	0.000***↓	0.001**↑	0.417	0.041*	0.171	0.762
Glomeraceae	0.87 ± 0.02	0.019*↑	0.456	0.000***↓	0.101	0.828	0.805	0.376
Paraglomeraceae	0.01 ± 0.00	0.008**↓	0.000***↓	0.026*↑	0.003**	0.198	0.260	0.098 ^.^
Edaphophilic guild	0.00 ± 0.00	0.006**↓	0.845	0.012*↑	0.513	0.098 ^.^	0.901	0.236
Diversisporaceae	0.00 ± 0.00	0.917	0.434	0.086^.^↑	0.335	0.638	0.922	0.118
Gigasporaceae	0.00 ± 0.00	0.000***↓	0.788	0.144	0.791	0.277	0.749	0.445
Ancestral guild	0.09 ± 0.02	0.255	0.542	0.000***↑	0.139	0.661	0.401	0.931
Acaulosporaceae	0.01 ± 0.00	0.004**↑	0.816	0.811	0.781	0.722	0.894	0.988
Ambisporaceae	0.07 ± 0.02	0.072^.^↓	0.464	0.002**↑	0.326	0.976	0.562	0.652
Archaeosporaceae	0.01 ± 0.00	0.566	0.135	0.000***↑	0.150	0.657	0.974	0.469

*, *P* < 0.05; **, *P* < 0.01; ***, *P* < 0.001;, *P* < 0.1; ↑, positive main effect; ↓, negative main effect. *P* values from a three‐way ANOVA are shown using N, P and K deficiency (−N, −P, −K) as factors, including testing their interactions (−N*−P, −N*−K, −P*−K, −N*−P*−K). The effects of N, P, and K deficiencies on soil and root AMF community compositions are analysed by PERMANOVA. The analysis is based on a fully factorial design of N, P and K deficiency with four replicate plots per treatment (*n* = 4, total nr of plots: 32). Relative abundances of guilds and families are presented as the mean across all inorganic treatments (±SE, *n* = 32). Bold values show *P* below 0.05 and the nutrients in the header represent deficiency.

**Fig. 2:**
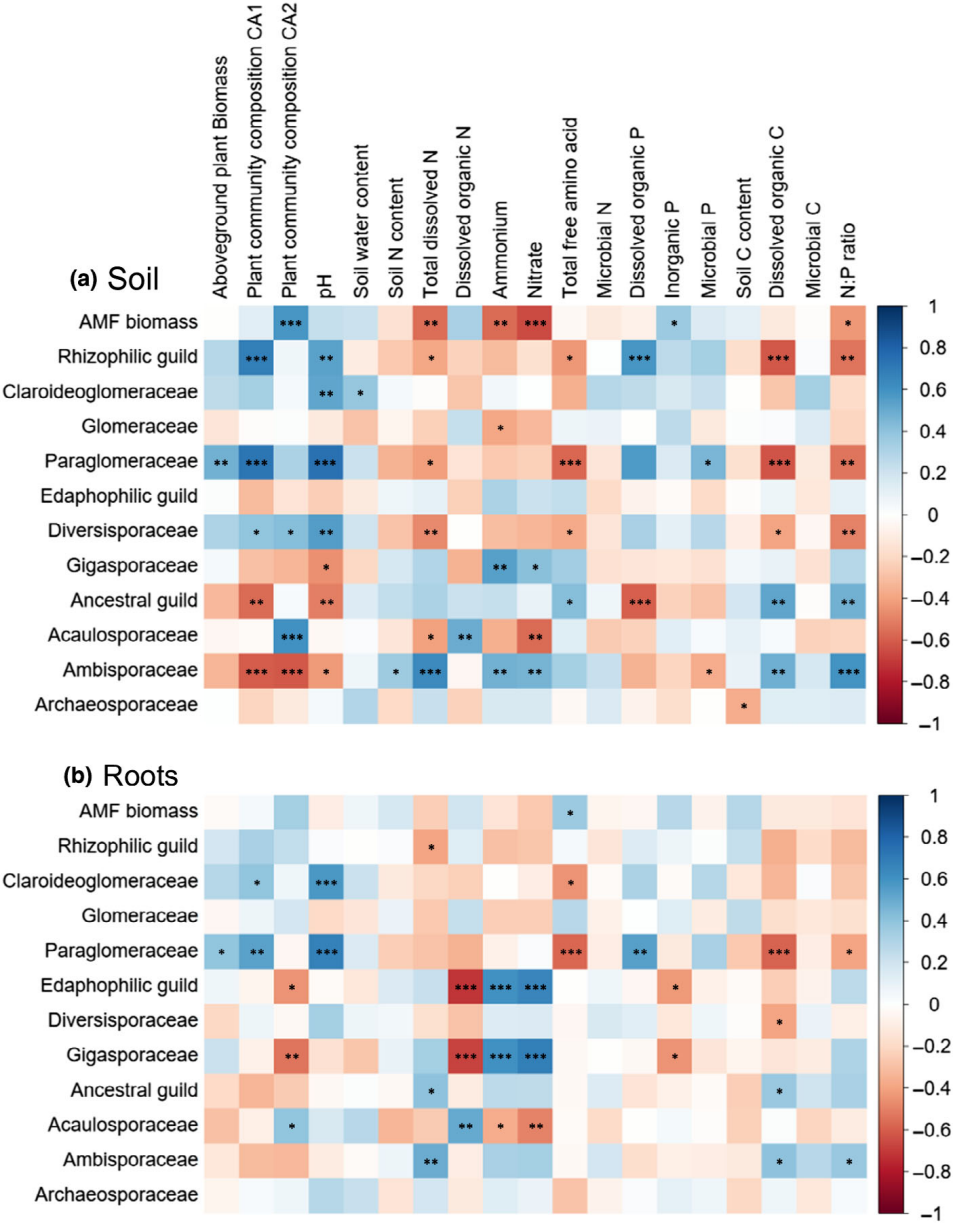
Soil (a) and root (b) arbuscular mycorrhizal fungi (AMF) biomass, guilds and families are correlated with plant and soil properties across nitrogen (N), phosphorus (P) or potassium (K) deficiency treatments. Colours in the correlation heatmap show negative (red) and positive (blue) associations based on Spearman's correlation coefficients. The intensity of colours indicates the strength of the correlations, and the stars represent the statistical significance: *, *P* < 0.05; **, *P* < 0.01; ***, *P* < 0.001. Correlation analysis was based on inorganically fertilized treatments only (including the unfertilized control). For correlation analysis among all treatments, please see Supporting Information Fig. S8, and for Spearman's correlation coefficients, refer to Fig. S13. Moreover, Fig. S12 shows correlations between soil and root AMF genera and environmental parameters across inorganic treatments.

Treatment effects on AMF biomass were also reflected in correlations of soil and root AMF biomass with soil N and P pools. Soil AMF biomass was negatively correlated with dissolved inorganic N pools (ammonium and nitrate) and the ratio of dissolved N to dissolved P, while root AMF biomass showed no correlation with soil N or P pools (but note that K pools were not measured in this study) (Fig. 2).

While the fully inorganically fertilized (NPK) treatment decreased AMF biomass, organic fertilization increased AMF biomass in soils (but not in roots) (Fig. S4). Solid manure increased soil AMF biomass (*F* = 6.11, *P* < 0.05), but its combination with liquid slurry lessened this effect (*F* = 8.50, *P* < 0.05; Fig. S4a).

### AMF community composition differed systematically between soils and roots

**Fig. 3:**
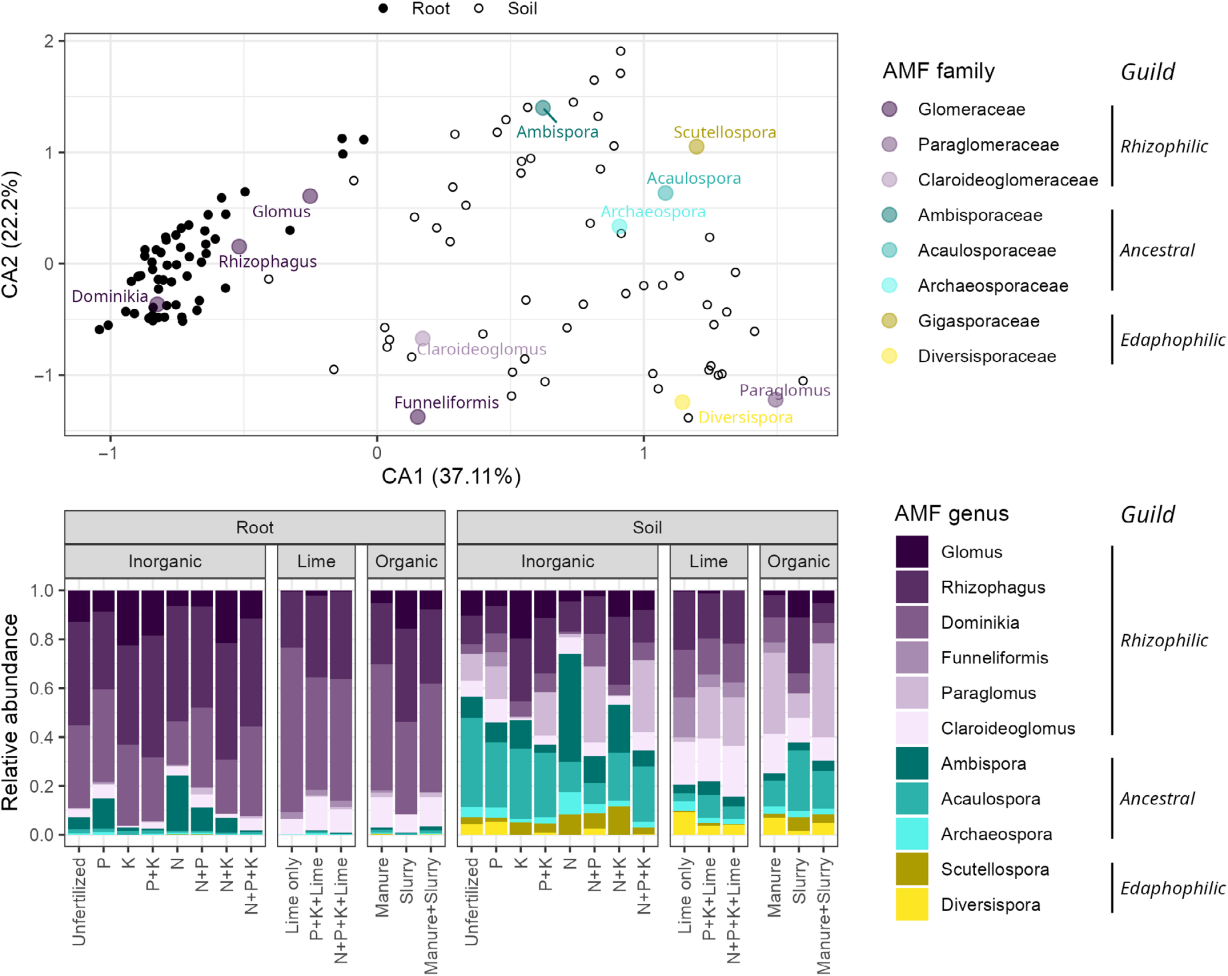
Arbuscular mycorrhizal fungi (AMF) community compositions differed systematically between soils and roots and were shaped by long‐term fertilization regimes. Root samples were significantly separated from soil samples in a multivariate correspondence analysis (CA) based on AMF genera (upper panel). Shown is a biplot of the first two CA axes, which together explain 59% of the total variability of the data. Small black‐and‐white circles represent soil and root samples (open circles, soil; closed circles, root). Larger coloured circles indicate positions of AMF genera on the biplot, with colours indicating their families. Information on treatments associated with each data point in the CA biplot can be found in Supporting Information Fig. S5. The lower panel shows mean relative abundances of soil and root AMF genera for each long‐term fertilization regime (*n* = 4). The relative abundances of rhizophilic, ancestral and edaphophilic guilds are depicted in purple, cyan and yellow, respectively. Data shown in this figure are based on 18S rRNA gene amplicon sequencing using RNA (cDNA) for soil, and DNA for root samples (a comparison of soil DNA with root DNA data yielded similar results and can be found in Fig. S2).

In both soil and root samples, we found 11 AMF genera from eight families (Fig. 3). Roots were dominated (*c*. 75–99%) by AMF families and genera of the rhizophilic guild (mostly *Dominikia*, *Rhizophagus* and *Glomus*; Fig. 3). By contrast, soil samples contained substantial proportions (*c*. 20–75%) of ancestral and edaphophilic taxa, particularly in inorganically fertilized plots (Fig. 3).

Root samples were significantly separated from soil samples in a multivariate CA over all treatments (PERMANOVA: *F* = 81.35, *P* < 0.001; Fig. 3). *Rhizophagus*, *Dominikia* and *Glomus* (all Glomeraceae) were more abundant in roots, while *Scutellospora* and *Acaulospora* (belonging to edaphophilic and ancestral guilds, respectively) were more abundant in soils (Fig. 3). Although *Claroideoglomus*, *Funneliformis* and *Paraglomus* are classified as rhizophilic AMF by Weber *et al*. (2019), they were more abundant in soil samples in our experiment and grouped closer to edaphophilic AMF (*Diversispora* and *Scutellospora*) along the CA1 axis, which separated roots from soil samples. Additionally, the two edaphophilic families were clearly separated along the CA2 axis, which reflected the variability of the fertilization treatments (Fig. S5). For example, *Scutellospora* clustered with ancestral AMF along CA2, as both exhibited a relatively higher abundance at unfertilized and N‐ and/or K‐fertilized plots. Diversispora, on the other hand, clustered with *Paraglomus*, *Claroideoglomus* and *Funneliformis* along the CA2 axis, as all were relatively more abundant at organic fertilization treatments (Fig. S5).

This separation of root and soil samples was also visible when comparing soil and root DNA (Fig. S2), which rules out that it could have been caused by differences between DNA and RNA abundances in roots and soil, respectively.

### AMF community composition is associated with N and P in soils, but with K in roots

While the axis with the largest explanatory power separates root from soil samples (CA1, explaining 37.11% of the variability), the second axis (CA2, explaining 22.2% of the variability) is driven by the fertilization treatments (Figs 3, S5). A clear separation exists among lime, organically fertilized and the inorganically fertilized plots, which is consistent across root and soil AMF communities (PERMANOVA: *F* = 8.90, *P* < 0.001; Fig. S5). Separate multivariate analyses of root and soil communities (inorganic treatments only) revealed that soil AMF composition was mostly affected by N and P, and less by K deficiencies, while root AMF communities were significantly affected only by K deficiency (PERMANOVA; Fig. S6; Table 1). This pattern, with N and P affecting soil AMF and K affecting root AMF communities, aligns with our AMF biomass findings (Fig. 1; Table 1).

### Distinct response of AMF guilds and families to inorganic nutrient deficiencies

**Fig. 4:**
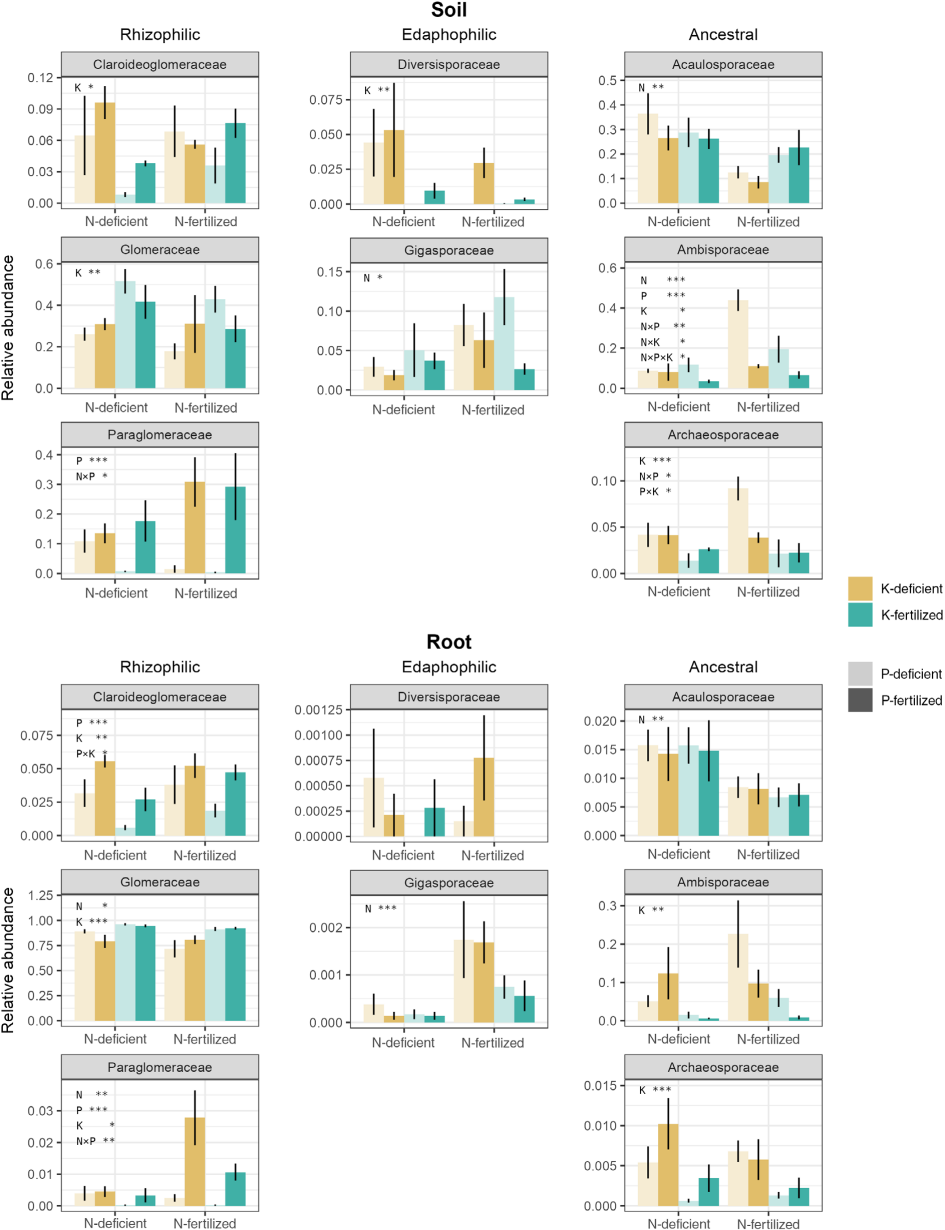
Arbuscular mycorrhizal fungi (AMF) families showed distinct responses to long‐term deficiencies of nitrogen (N), phosphorus (P) or potassium (K), which are partly consistent across soil (upper panel) and roots (lower panel). AMF families were grouped into exploration‐based functional guilds (rhizophilic, edaphophilic and ancestral) according to Weber *et al*. (2019). Barplots show relative abundances of each AMF family on the total AMF. X‐axes depict N treatments (left bars, N‐deficient; right bars, N‐fertilized). Colours indicate K treatments (ochre, K‐deficien; cyan, K‐fertilized). Colour transparency indicates P treatments (light colours, P‐deficient; dark colours, P‐fertilized). Bars represent mean ± SE of the mean (*n* = 4). Statistical significance from three‐way analysis of variance is stated within each panel: *, *P* < 0.05; **, *P* < 0.01; ***, *P* < 0.001 (Table 1).

We observed strong and clear responses of AMF families and guilds to nutrient deficiencies, which were mostly consistent across soil and roots. For example, P‐deficient plots showed lower relative abundances of Paraglomeraceae (in roots and soil) and Claroideoglomeraceae (in roots), both belonging to the rhizophilic guild (*P* < 0.001; Fig. 4; Table 1). At the guild level, the rhizophilic guild declined in P‐deficient plots in soil, while the ancestral guild increased (Fig. S7), the latter mostly driven by an increase in Ambisporaceae (*P* < 0.001; Fig. 4; Table 1).

By contrast, N deficiency did not affect rhizophilic guild members but decreased Gigasporaceae (edaphophilic guild) in both sample types (Figs 4, S7; Table 1). Moreover, N deficiency shifted families within the ancestral guild, increasing Acaulosporaceae and decreasing Ambisporaceae in soil, with similar trends in roots (Fig. 4; Table 1).

While P deficiency reduced less dominant rhizophilic families such as Paraglomeraceae and Claroideoglomeraceae, K deficiency negatively affected the dominant rhizophilic family Glomeraceae in both roots and soils (Fig. 4; Table 1), shaping a significantly negative response of the rhizophilic guild to K deficiency (Fig. S7; Table 1). Simultaneously, K deficiency increased ancestral families, mainly Archaeosporaceae and Ambisporaceae in roots and soils, and the edaphophilic guild in roots (Figs 4, S7; Table 1).

The significant effect of long‐term deficiencies and imbalances was also reflected in correlations between the relative abundance of families and soil N and P pools (Fig. 2). For instance, Gigasporaceae and Ambisporaceae positively correlated with ammonium and nitrate, while Acaulosporaceae showed a significant negative correlation with nitrate. Moreover, Paraglomeraceae correlated positively with dissolved organic P, while the ancestral guild was negatively associated with it in soil. Relationships between AMF families and soil N and P pools strengthened when considering all treatments (Fig. S8).

### Changes in soil AMF communities were linked to changes in plant communities

Long‐term nutrient deficiencies influenced plant communities. A CA showed plant community composition was influenced by all three elemental deficiencies, with N and P deficiencies being more significant (PERMANOVA: *P* < 0.001) than K deficiency (PERMANOVA: *P* < 0.05; Fig. S9).

**Fig. 5:**
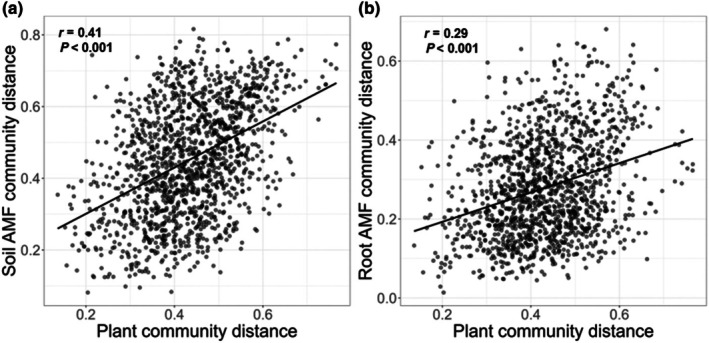
Stronger association of plant communities with soil than with root arbuscular mycorrhizal fungi (AMF) communities. Mantel test results show correlations between (a) plant and soil AMF communities, and (b) plant and root‐associated AMF community compositions across all treatments (including the unfertilized, inorganic, lime and organic treatments). Each dot represents a pairwise comparison of community dissimilarities (Bray–Curtis distances) between plots. *r* indicates Spearman's rank correlation coefficient between the distance matrices. For the association between soil and root AMF and plant communities by Mantel test among inorganic treatments, please see Supporting Information Fig. S10.

Plant community composition correlated significantly with soil AMF community composition, and less with root AMF community composition, as shown by a Mantel test (Figs 5, S10). This was evident across all treatments (*r* = 0.41, *P* < 0.001 and *r* = 0.29, *P* < 0.001 for soil and root AMF, respectively; Fig. 5), but remained significant for soil AMF even in inorganic treatments (*r* = 0.28, *P* < 0.001 and r = 0.09, *P* > 0.05 for soil and root AMF, respectively; Fig. S10). When controlling for pH and dissolved inorganic N, which were identified via CCA as the strongest environmental drivers of both AMF and plant community compositions, in a partial Mantel test, correlations were reduced but mostly remained significant (All treatments: *r* = 0.26, *P* < 0.001 and *r* = 0.10, *P* < 0.05 for soil and root AMF, respectively; inorganic treatments: *r* = 0.19, *P* < 0.001 for soil AMF and not significant for root AMF).

### Link between soil properties, and plant and AMF communities

We tested the link between soil properties and community composition of AMF and plants using CCA (Tables 2, S2). pH and dissolved inorganic N correlated significantly with root and soil AMF communities across inorganic treatments (excluding lime plots; Table 2). Soil AMF communities were, as already indicated by the Mantel test, additionally linked to plant community composition (using the first correspondence axis of plant CA as a predictor; Table 2). Like AMF communities, plant community composition was most linked to not only pH and dissolved inorganic N but also inorganic P. When all treatments were considered, these relationships remained, and additional predictors emerged: dissolved organic N, P and aboveground biomass for soil AMF; total free amino acids for root AMF; and soil total N for plants (Table S2).

**Table 2:** Effect of environmental variables on soil and root arbuscular mycorrhizal fungi (AMF), and plant community compositions across the inorganic treatments by canonical correspondence analysis (CCA).

Environmental factors	df	χ^2^	*F*‐value	*P*‐value
Soil AMF				
pH	**1**	**0.1425**	**7.54**	0.000***
Dissolved inorganic N	**1**	**0.0869**	**4.6**	0.000***
Plant community composition CA1	**1**	**0.0568**	**3**	0.009**
Soil water content	1	0.0319	1.69	0.125
Plant community composition CA2	1	0.0242	1.28	0.255
Dissolved organic C	1	0.0217	1.15	0.329
Root AMF				
Dissolved inorganic N	**1**	**0.0729**	**9.08**	0.000***
pH	**1**	**0.0331**	**4.11**	0.009**
Total free amino acid	1	0.0174	2.17	0.086^.^
Plant community composition CA1	1	0.0136	1.7	0.156
Inorganic P	1	0.0084	1.05	0.373
Dissolved organic C	1	0.0069	0.85	0.471
Plant				
pH	**1**	**0.078**	**2.59**	0.000***
Dissolved inorganic N	**1**	**0.0704**	**2.33**	0.000***
Inorganic P	**1**	**0.0605**	**2.01**	0.004**
Dissolved organic P	**1**	**0.0483**	**1.6**	0.031*
Dissolved organic C	1	0.042	1.39	0.094^.^
Soil water content	1	0.0412	1.37	0.102

*, *P* < 0.05; **, *P* < 0.01; ***, *P* < 0.001; ^.^, *P* < 0.1. Significance of each variable was assessed using permutation tests (*n* = 9999). Environmental variables with a variance inflation factor (VIF) < 10 were selected to avoid the effects of multicollinearity. Tested variables were pH, dissolved inorganic N, dissolved organic P, inorganic P, soil water content, total free amino acids, soil N content, dissolved organic C, plant community composition CA1, CA2 and above ground plant biomass. Plant community composition CA1, CA2 and above ground plant biomass were not tested for plant community composition. Variables with a VIF > 10 such as total dissolved N, dissolved organic N, soil C content, N : P ratio, and microbial C, N and P were filtered. Shown are the six most significant factors for each community; bold environmental factors show *P* below 0.05. For the analysis among all treatments, please see Supporting Information Table S2.

pH also correlated strongly with the relative abundances of AMF guilds and families across inorganic (Fig. 2) and all treatments (Fig. S8). It positively correlated with rhizophilic AMF, particularly Paraglomeraceae in soils and roots. By contrast, pH negatively correlated with the ancestral guild (particularly Ambisporaceae) and the family Gigasporaceae (edaphophilic guild) in soil samples (Fig. 2).

Liming substantially increased soil pH values (up to 6.5) and soil AMF abundance (*F* = 86.33, *P* < 0.001), especially in limed, but unfertilized plots where AMF biomass almost doubled (Fig. S11a). The positive effect of liming on soil AMF decreased when PK or NPK fertilizers were added (*P* < 0.01 for both PK + Lime and NPK + Lime compared to PK and NPK treatments, respectively). The effect of liming on root AMF was smaller, but still significant (*F* = 6.90, *P* < 0.05; Fig. S11c).

## Discussion

Prolonged soil nutrient deficiencies disrupt the balance among plants, their AMF partners and soil nutrient states. Predicting the outcomes of such disruptions is challenging due to the complexity of such interactions. Our findings show that 70 yr of nutrient removal through biomass harvests significantly affected AMF communities in managed grasslands. Distinct fungal families responded differently to specific nutrient deficiencies, and these community shifts were linked to changes in plant composition and soil properties.

### Strong interplay between N, P and K determines soil AMF abundance and fungi‐to‐plant biomass ratios

AMF are considered crucial for P nutrition of grassland plants (Smith & Smith, 2011). Although the role of AMF in N nutrition is less established, increasing evidence demonstrates that AMF contribute significantly to N acquisition (Thirkell *et al*., 2016; Munene *et al*., 2025). We thus hypothesized that P availability has a greater impact on AMF communities than on N. Contrary to this, however, N played the most important role for soil AMF biomass, with more AMF present in N‐deficient than in N‐fertilized soils. P deficiency showed an opposing trend by decreasing soil AMF biomass (*P* = 0.09), but only when K was also deficient. Both N and P deficiencies significantly increased the ratio of soil AMF‐to‐plant biomass in plots receiving K fertilization. This was driven by a decline in plant growth, while AMF biomass remained steady (Fig. 1). In K‐deficient plots, plant biomass was less affected by further N or P deficiency, but the positive impacts of N deficiency on AMF biomass were stronger. These results suggest that plant P or N limitation in K‐fertilized plots may have led to higher plant support of AMF per unit of (aboveground) plant biomass, as has been suggested for P limitation (Beauregard *et al*., 2010; Propster & Johnson, 2015; Hu *et al*., 2019). However, this additional support under P limitation seems not to have been maintained under K colimitation. K has been shown to enhance plant P uptake via both root and mycorrhizal pathways (Han *et al*., 2023) and a strong link between P and K has been demonstrated in mycorrhizas, suggesting a K and P interaction during mycelial transport (Garcia & Zimmermann, 2014). K limitation may thus constrain plant P uptake through the mycorrhizal symbiosis, explaining the lack of response of the soil AMF/plant biomass ratio to P deficiency in K‐limited soils (Fig. 1).

Stoichiometry‐based concepts, such as ‘trade balance’ and ‘functional equilibrium’ models, suggest AMF symbiosis functionality depends on the N‐to‐P ratio (Johnson, 2010). If P is not added alongside N, increased N availability leads to aggravation of relative P deficiency in plants, prompting plants to allocate more C to AMF to improve P uptake. Conversely, when P is co‐abundant, plants reduce C allocation to AMF in favour of aboveground structures. Thus, it has been suggested that AMF biomass increases with N when P is limiting, but decreases when it is abundant (Johnson, 2010; Wipf *et al*., 2019). By contrast, we found no direct interaction between N and P in soil AMF biomass or AMF : plant biomass ratio, but strong interactions between either N or P addition and the K manipulation treatment (Fig. 1; Table 1). Our results demonstrate that K fertilization or the lack thereof plays an important and possibly so far overlooked role in shaping AMF responses to varying nutrient availabilities.

### Lack of K fertilization strongly decreased root colonisation by AMF

Unlike soil AMF, which were predominantly influenced by N, root AMF were mainly affected by K, with a notable decrease in biomass under K deficiency (Fig. 1; Table 1). Previous studies, though limited, support a positive link between K and AMF root colonization (Liu *et al*., 2019; Han *et al*., 2023).

K is essential for plant functions such as carbon and water transport (by facilitating plant transpiration via stomata regulation), photosynthesis, nutrient uptake and stress response (Cakmak, 2005; Egilla *et al*., 2005; Wang & Wu, 2013; Sardans & Peñuelas, 2015). Particularly, K facilitates saccharide transfer from leaf parenchymal cells to the phloem (Deeken *et al*., 2002; Zörb *et al*., 2014; Sardans & Peñuelas, 2021) and is key for supplying sink organs with photosynthates (Zörb *et al*., 2014). K deficiency reduces photosynthetic CO_2_ fixation and impairs transport of photoassimilates to belowground plant organs (Cakmak, 2005), which could explain the 50% reduction in root AMF biomass of K‐deficient plots and the lack of positive AMF response to P deficiency. In addition, K deficiency increases NADPH oxidase activity, leading to reactive oxygen species accumulation in roots (Cakmak, 2005; Garcia *et al*., 2017), which could damage plant cell membranes and AMF in close proximity, creating unfavourable conditions for colonisation.

Impaired water transport in plants may also help to explain the significantly increased soil water content we observed in K‐deficient plots (Fig. S3b; Table S1). Although soil moisture could have been influenced by many different factors, we did not find any correlation except for the K treatments. As water content was, however, not related to AMF biomass in soils or roots (Fig. 2), we assume that enhanced water contents did not directly affect AMF biomass.

Notably, the impact of K deficiency was strongest on Glomeraceae AMF, which dominated in roots but were less abundant in soils (Fig. 4; Table 1). This might explain the more significant effect of K deficiency on root AMF vs soil AMF. While we cannot, with existing knowledge, explain why Glomeraceae would be more susceptible to K deficiency, we can speculate that soil‐based AMF families may have benefited more from altered soil properties such as increased soil pH or water content in K‐deficient plots, while root‐centred Glomeraceae were more affected by disturbed plant physiological processes due to K deprivation.

### Proportions of rhizophilic, edaphophilic and ancestral guilds differed between root and soil samples

The rhizophilic guild (particularly Glomeraceae) was relatively more abundant in roots, while ancestral and edaphophilic guilds predominated in soil (Fig. 3). For edapho‐ and rhizophilic AMF, this reflects their exploration traits: Rhizophilic AMF produce a relatively higher proportion of hyphae inside roots, while edaphophilic AMF produce relatively more extraradical hyphae (Powell *et al*., 2009; Weber *et al*., 2019). Nevertheless, our results also indicate notable variation within the guild classification proposed by Weber *et al*. (2019) (Fig. 3). Within the Glomeraceae, *Funneliformis* exhibited comparatively higher abundance in soil than in roots, aligning more closely with edaphophilic tendencies than with the classically rhizophilic profile of other Glomeraceae. Similarly, Claroideoglomeraceae and Paraglomeraceae were more abundant in soil than in roots (Fig. 3), a pattern that is also more consistent with edaphophilic guilds. These responses suggest that some lineages traditionally classified as rhizophilic may express broader or more flexible exploration strategies than expected.

Although ancestral AMF are thought to produce low amounts of both extra‐ and intraradical hyphae (Powell *et al*., 2009; Weber *et al*., 2019), we found them in surprisingly high proportions in both soil and roots, exceeding even edaphophilic AMF in soils (Fig. 3). This could indicate that ancestral AMF produce more hyphae than previously thought or suggest a higher number of ancestral than edaphophilic individuals. Interestingly, some ancestral families (Acaulosporaceae and Archaeosporaceae) were relatively more abundant in soils than roots (Fig. 3), suggesting they may exhibit more ‘edaphophilic’ exploration traits than commonly assumed. This, together with our findings about their specific response to nutrient limitations, indicates that ancestral AMF may possess unique functional traits and may play more important ecosystem roles than previously thought (Maherali & Klironomos, 2007).

### AMF functional groups and families show highly specific responses to nutrient deficiencies

Although root and soil AMF biomass responded differently to nutrient deficiencies, responses of individual AMF families and guilds remained consistent across both sample types (Table 1). The specific and significant responses of individual AMF families to nutrient deficiencies suggest that soil nutrient conditions exerted strong selection pressure at the family level. Although we grouped AMF families into exploration‐based functional guilds (Weber *et al*., 2019), contrasting responses within guilds suggest that additional functional traits at the family level shape responses to nutrient limitations.

While little is known about other AMF functional traits at the family level, exploration traits have been used to explain family‐ and guild‐level responses to nutrient availability (Treseder *et al*., 2018; Weber *et al*., 2019; Han *et al*., 2020). Some reviews proposed that AMF with high soil exploration capabilities, such as Gigasporaceae, decline in N‐enriched soils due to reduced plant C allocation (Cotton, 2018; Treseder *et al*., 2018; Lilleskov *et al*., 2019), while AMF with limited soil exploration capabilities, such as Glomeraceae, might be favoured (Treseder *et al*., 2018), possibly due to increased plant pathogen pressure at high N availability (Sun *et al*., 2020). This contrasts with the trade‐balance model, which predicts plant preference for high‐exploration AMF under N enrichment to compensate for relative P limitation (Johnson, 2010).

Our results oppose the concept that N fertilization reduces the fraction of edaphophilic taxa. Although fewer AMF could thrive in N‐fertilized soil, the relative abundance of Gigasporaceae (edaphophilic guild) increased in soil and roots (Fig. 4; Table 1). In line with a global‐scale meta‐analysis showing that N addition decreased Glomeraceae abundance while minimally affecting Gigasporaceae (Han *et al*., 2020), we observed a negative effect of N addition on Glomeraceae, although this was significant only in roots (Fig. 4; Table 1). Interestingly, the ancestral family Acaulosporaceae, which showed an ‘edaphophilic’‐like hyphal distribution, responded positively to N deficiency, contrasting with Gigasporaceae. However, we have to note that our amplicon sequencing‐based data reflect only relative shifts among AMF families. As N deficiency increased total AMF biomass (NLFA 16:1ω5), it is possible that Gigasporaceae remained constant in absolute terms, while Acaulosporaceae increased both relatively and absolutely. The contrasting behaviour of Gigasporaceae and Acaulosporaceae is also demonstrated by Gigasporaceae correlating positively with inorganic N pools and negatively to organic N, while Acaulosporaceae showed the opposite pattern (particularly in roots; Fig. 2). Why N deficiency favours Acaulosporaceae over Gigasporaceae remains unclear. Based on their contrasting correlations to inorganic and organic dissolved N, we speculate that Acaulosporaceae might be more effective at N acquisition when mineral N is limiting, but dissolved organic N is abundant, which is the situation we observed in the N deficiency treatments (Fig. S3; Table S1). However, direct measurements of plant N uptake through these AMF families remain lacking and should be pursued in future studies.

Contrary to N, which mostly affected edaphophilic and ancestral guilds, P and K primarily affected rhizophilic families (Fig. 4; Table 1). While K deficiency strongly decreased Glomeraceae, P deficiency significantly decreased Paraglomeraceae in both soil and roots, and Claroideoglomeraceae in roots. Correspondingly, Paraglomeraceae showed the strongest positive correlation with dissolved organic P, followed by Claroideoglomeraceae (Fig. 2). While previous studies revealed a positive link between P and Paraglomeraceae (Kurle & Pfleger, 1996; Hijri *et al*., 2006), others found no such association (Gosling *et al*., 2014). Both families, particularly Paraglomeraceae, however, showed the strongest correlation among all families with soil pH (Fig. 2). As the P fertilizer contained alkaline additions, which increased soil pH, the increase in Paraglomeraceae and Claroideoglomeraceae could thus also reflect a preference for higher pH, as commonly observed for AMF (Helgason & Fitter, 2009; Han *et al*., 2020).

The negative impact of P deficiency on Paraglomeraceae contrasted with its positive effect on Ambisporaceae (ancestral guild) (Fig. 4; Table 1). Ambisporaceae were positively correlated with ammonium, nitrate and N : P ratio, and negatively with pH in soil samples (Fig. 2). Together, our results suggest contrasting functional roles between certain rhizophilic (especially Paraglomeraceae) and ancestral (particularly Ambisporaceae) families regarding P availability. Notably, Paraglomeraceae and Ambisporaceae were strongly associated with plant community composition in opposite directions (Fig. 2). This indicates that their switch with P deficiency was linked to plant community changes, suggesting a certain degree of host preference. Interestingly, Ambisporaceae showed the strongest positive response to P deficiency, surpassing Gigasporaceae (Table 1), a commonly studied family under nutrient limitation (Powell *et al*., 2009; Treseder *et al*., 2018). We hypothesize that Ambisporaceae might play a critical yet underappreciated role in P uptake from P‐limited soils.

Similar to P deficiency, K deficiency shifts the community from rhizophilic families to ancestral and, to some extent, edaphophilic families. K deficiency decreased the dominant rhizophilic family, Glomeraceae, while increasing Archaeosporaceae and Ambisporaceae (ancestral guilds), and Diversisporaceae (edaphophilic guild) (Fig. 4; Table 1). This shift may reflect plant reliance on ancestral and edaphophilic AMF with extensive extraradical mycelia for P and K uptake under limiting conditions. Like P, K is relatively immobile in soil due to strong adsorption to particles, leading to root depletion zones. AMF could thus be essential for plant K uptake. The influence of mycorrhiza formation on plant K nutrition remains poorly understood (Sardans *et al*., 2023). Although several studies showed that AMF enhanced plant K uptake under controlled conditions (Garcia & Zimmermann, 2014; Garcia *et al*., 2017; Kafle *et al*., 2022; Han *et al*., 2023, 2025; Alizadeh *et al*., 2025), inoculating soybeans with AMF did not enhance K uptake under field conditions (Cooney *et al*., 2025). Our results indicate that Archaeosporaceae, Ambisporaceae and Diversisporaceae might possess traits supporting plant K uptake under K‐limiting conditions, or provide adaptation mechanisms to host plants under nutrient stress (Garcia *et al*., 2017).

Noteworthy, we observed contrasting family‐level responses to specific nutrient deficiencies within each exploration‐trait‐based guild. While Ambisporaceae and, to a lesser extent, Gigasporaceae may support plants under P deficiency, Archaeasporaceae, Ambisporaceae and the Diversisporaceae seem to play a critical role under K deficiency. Notably, N deficiency uniquely increased Acaulosporaceae (ancestral guild). Our analysis indicates that families with specific and complementary nutrient specializations exist within each guild, particularly high nutrient deficiency competence within the ancestral guild, whose families often behaved antagonistically to families of other guilds. Investigating functional properties of understudied AMF families will help expand the concept of AMF functional groups and enable targeted exploration of plants with specific AMF to reduce fertilizer use.

### AMF were closely linked to plant communities and soil pH

AMF are not host‐specific (Klironomos, 2000) but exhibit host preference (Vandenkoornhuyse *et al*., 2002; Martínez‐García *et al*., 2015). Different plant functional groups select distinct AMF communities, potentially based on fungal life‐history traits (Chagnon *et al*., 2013; Davison *et al*., 2020; Blažková *et al*., 2021). While these factors may explain the link between plant and soil AMF communities, both could also have responded similarly to changing soil parameters. Research showed that soil conditions were more influential in shaping AMF communities in European grasslands than host plant specificity (Van Geel *et al*., 2018). Edaphic factors may exert stronger costructuring effects on the soil‐dominant AMF proportion, as extraradical hyphae are more exposed to edaphic parameters than intraradical hyphae. This could explain the stronger correlations between plant and soil AMF communities than between plant and root AMF communities (Fig. 5). Among all edaphic factors, soil pH showed the strongest link to both plant and AMF communities (Tables 2, S2). AMF prefer neutral or alkaline soil pH (Helgason & Fitter, 2009; Han *et al*., 2020), explaining higher AMF biomass in limed plots, particularly in soil (Fig. S11). The absence of liming kept pH within a moderately acidic range, possibly accounting for the lack of correlation between AMF biomass and pH in inorganic treatments (Fig. 2). However, pH remained the best explanator for soil AMF community composition even across inorganic treatments (Table 2), confirming previous findings highlighting narrow ecological niches for AMF, particularly for pH (Kawahara *et al*., 2016; Davison *et al*., 2021). The strong correlations between soil pH and both AMF and plant community compositions suggest strong interdependencies.

Interestingly, while topsoil C content significantly varied with fertilization regimes (Table S1), it did not correlate with AMF families, guilds or biomass (Figs 2, S8), despite significant shifts across exploration‐based guilds (Fig. S7). Although AMF guilds are thought to affect soil C stocks through different mycelial structures and biomass (Horsch *et al*., 2023), we conclude that this may not be pronounced in agricultural settings, in which fertilization‐driven changes in decomposition and fine root biomass likely overrule the effect of AMF functional group distribution.

### Conclusion

Our research shows that N, P and K deficiencies caused by biomass harvest without adequate element replacement significantly alter AMF biomass and communities. Prolonged K deficiency, a relatively understudied factor, greatly affects AMF and modulates the N and P effects. Notably, the combination of N fertilization with inadequate K fertilization, widespread in global agriculture (Manning, 2012; Tan *et al*., 2012; Zörb *et al*., 2014), may be most detrimental for AMF biomass and root colonisation, and cause strong shifts in AMF families and guilds, with yet unknown effects on ecosystem functioning.

We reveal specific and complementary nutrient specializations at the AMF family level within exploration‐based guilds. Understanding these responses could help improve agricultural AMF inoculation strategies, which currently focus primarily on Glomeraceae taxa (Zhang *et al*., 2019; Lutz *et al*., 2023). Our study highlights the need to better understand the diverse spectrum of AMF families, especially ancestral ones, which is only possible through molecular approaches that overcome traditional biases towards Glomeraceae.

These findings highlight the intricate role of K alongside N and P in shaping AMF community structure and function. Moving beyond model taxa, our results advocate for incorporating diverse AMF families with complementary nutrient specializations into agricultural management, fostering resilient and efficient symbioses that are able to address widespread nutrient imbalances.

## Competing interests

None declared.

## Author contributions

KJ was involved in conceptualization, formal analysis, investigation, visualization. LA was involved in formal analysis. KG was involved in formal analysis, visualization, methodology. SG was involved in formal analysis, investigation, visualization. SD was involved in investigation. LF was involved in formal analysis, investigation. AC was involved in formal analysis, investigation. VM, JW, FS, BI and BI were involved in investigation. KH‐A was involved in conceptualization. EMP was involved in funding acquisition, project administration, resources. AR was involved in conceptualization, funding acquisition, project administration, supervision, resources. JJ was involved in conceptualization, formal analysis, investigation, methodology, supervision. CK was involved in conceptualization, funding acquisition, project administration, supervision, validation. KJ and CK wrote the original draft and all other co‐authors contributed to review and editing.

## Disclaimer

The New Phytologist Foundation remains neutral with regard to jurisdictional claims in maps and in any institutional affiliations.

## Supporting information


**Fig. S1** Experimental plot setup.
**Fig. S2** The effects of long‐term nutrient deficiencies on soil (DNA‐based) and root (DNA‐based) AMF communities.
**Fig. S3** The impact of long‐term nutrient deficiencies on soil properties and nutrient content.
**Fig. S4** Effects of long‐term organic fertilization (Manure and slurry) on soil and root AMF biomass.
**Fig. S5** Compositions of AMF communities in combined soil and root samples exposed to different long‐term nutrient deficiencies evaluated by correspondence analysis (CA).
**Fig. S6** The effects of long‐term nutrient deficiencies on soil and root AMF community composition based on correspondence analysis (CA).
**Fig. S7** The effects of long‐term deficiencies of N, P and K on soil and root AMF functional guilds.
**Fig. S8** Relationship between AMF and environmental parameters across all treatments.
**Fig. S9** The impacts of long‐term nutrient deficiencies on plant community composition.
**Fig. S10** Relationship of plant communities with soil and root AMF communities across inorganic treatments.
**Fig. S11** The impact of long‐term lime application and fertilization regime on soil and root AMF biomass as well as the relationship of soil and root AMF biomass with pH.
**Fig. S12** Correlations between AMF genera and environmental factors.
**Fig. S13** Associations between AMF and environmental parameters across inorganic treatments.
**Table S1** Three‐way ANOVA results regarding the effect of long‐term deficiencies of N, P and K on edaphic factors in inorganic treatments.
**Table S2** Results of canonical correspondence analysis (CCA) testing the effect of environmental variables on soil and root AMF, and plant community compositions across all treatments.
Please note: Wiley is not responsible for the content or functionality of any Supporting Information supplied by the authors. Any queries (other than missing material) should be directed to the *New Phytologist* Central Office.

## Data Availability

Sequencing data are available through NCBI – GenBank under accession no. PRJNA1140020. All other data can be found in a public repository: doi: 10.5281/zenodo.13760726
